# Clinical and Histopathological Features of an Italian Monocentric Series of Primary Small Bowel T-Cell Lymphomas

**DOI:** 10.3390/cancers15102743

**Published:** 2023-05-13

**Authors:** Marco Lucioni, Sara Fraticelli, Giovanni Santacroce, Arturo Bonometti, Nicola Aronico, Roberta Sciarra, Marco Vincenzo Lenti, Paola Ilaria Bianchi, Giuseppe Neri, Monica Feltri, Benedetto Neri, Giuseppina Ferrario, Roberta Riboni, Gino Roberto Corazza, Alessandro Vanoli, Luca Arcaini, Marco Paulli, Antonio Di Sabatino

**Affiliations:** 1 Department of Molecular Medicine, University of Pavia, 27100 Pavia, Italy; 2 Pathology Unit, Fondazione IRCCS Policlinico S. Matteo, 27100 Pavia, Italy; 3 First Department of Internal Medicine, Fondazione IRCCS Policlinico S. Matteo, 27100 Pavia, Italy; 4 Department of Internal Medicine and Medical Therapeutics, University of Pavia, 27100 Pavia, Italy; 5 Pathology Unit, Humanitas Clinical and Research Center IRCCS, 20089 Rozzano, Italy; 6 Division of Haematology, Fondazione IRCCS Policlinico S. Matteo, 27100 Pavia, Italy; 7 Unit of Gastroenterology, Department of Systems Medicine, Tor Vergata University, 00133 Rome, Italy

**Keywords:** celiac disease, EATL, ITCL, ITCLDGT, lymphoproliferative disorders, MEITL, small bowel lymphoma

## Abstract

**Simple Summary:**

Intestinal T-cell lymphomas (ITCL) are a group of rare and very aggressive extranodal lymphomas classified into five subtypes. The current literature includes a small number of ITCL series from different countries, often focusing on a single ITCL subtype. In this study, we wanted to depict the complete clinical–pathological profile of a series of patients affected by primary ITCL involving the small bowel, diagnosed and treated at a referral center for celiac disease and lymphomas. Furthermore, we tried to define clinical and histopathological hallmarks of these lymphomas to widen the knowledge on these rare and still partially characterized neoplasms.

**Abstract:**

The gastrointestinal (GI) tract is the most common extranodal site of occurrence of non-Hodgkin lymphomas. Most GI lymphomas are of B-cell lineage, while T-cell lymphomas are less frequent. The aim of our retrospective study was to depict the clinical–pathological profile of a series of patients affected by intestinal T-cell lymphomas (ITCL) and possibly define hallmarks of these neoplasms. A total of 28 patients were included: 17 enteropathy-associated T-cell lymphomas (EATL), 5 monomorphic epitheliotropic T-cell lymphomas (MEITL), 3 indolent T-cell lymphoproliferative disorders of the gastrointestinal tract (ITCLDGT), and 3 intestinal T-cell lymphomas not otherwise specified (ITCL-NOS). Celiac disease (CD) was diagnosed in around 70% of cases. Diagnosis of EATL showed a significant correlation with CD30 expression, whereas MEITL with angiotropism and CD56 positivity. ITCLDGT cases showed plasma cells infiltration. Peripheral lymphocytosis, the absence of a previous diagnosis of CD, an advanced Lugano clinical stage, and the histological subtype ITCL-NOS were significantly associated with worse survival at multivariate analysis. Our findings about the epidemiological, clinical, and histopathological features of ITCL were in line with the current knowledge. Reliable prognostic tools for these neoplasms are still lacking but according to our results lymphocytosis, diagnosis of CD, Lugano clinical stage, and histological subtype should be considered for patient stratification.

## 1. Introduction

Extranodal lymphomas account for roughly one-third of all lymphoid malignancies. The gastrointestinal (GI) tract is the most common extranodal site involved in non-Hodgkin lymphomas. Between 20% and 40% of them are represented by primary gastrointestinal (GI) lymphomas as defined by the so-called Dowson’s criteria, i.e., (1) the presence of a bowel lesion, (2) the absence of peripheral lymphadenopathies, and (3) normal white blood cell counts [1,2,3,4]. Intestinal B-cell lymphomas greatly outnumber T-cell lymphomas, may involve the whole bowel length, and may be etiologically associated to several pathogens such as Helicobacter pylori and Hepatitis C virus [2,5]. On the other hand, intestinal lymphomas of T-cell origin are rarer and only a handful of entities are currently known, namely, intestinal T-cell lymphomas (ITCL), extranodal NK/T-cell lymphoma, nasal type (ENKTCL), and anaplastic large cell lymphoma (ALCL) [6,7,8].

According to the 2022 5th edition of the World Health Organization (WHO) classification, and the 2022 international consensus classification (ICC), ITCL are classified into five subtypes: enteropathy-associated T-cell lymphoma (EATL), monomorphic epitheliotropic ITCL (MEITL), indolent (clonal) T-cell lymphoproliferative disorder/lymphoma of the gastrointestinal tract (ITCLDGT), indolent NK-cell lymphoproliferative disorder of the gastrointestinal tract (iNKLPD), and ITCL not otherwise specified (NOS) [6,7,9,10].

EATL has the highest incidence among ITCL, is more common in Western countries, and arises in patients in their sixties or seventies. Its name relates to its association with celiac disease (CD) and Human Leucocyte Antigen (HLA)-DQ2 allele homozygosity [11]. EATL involves the small intestine in most cases, with multifocal lesions in a third to half of patients. Abdominal pain, B-symptoms, and diarrhea are the typical presenting symptoms. Common GI complications include hemorrhage, obstruction, and perforation [11]. Histologically, EATL shows a polymorphic infiltrate with medium- to large-sized cells intermingled with eosinophils and histiocytes [12]. EATL cells have a CD4/CD8 double-negative phenotype with expression of CD3, CD7, CD103, and at least partial expression of CD30 [13]. On a genetic level, EATLs are rearranged for the T-cell receptor (TCR)γ or TCRβ and display recurrent mutations in genes coding for JAK/STAT pathway molecules or histone-modifiers in most cases. The course of the disease is very aggressive, with a median overall survival of 7 months [11,13,14].

MEITL is rarer than EATL in our latitudes, but it shows a higher incidence in Asian and Hispanic populations. Clinically, MEITL displays many similarities with EATL but the association with CD is historically not recognized. MEITL infiltrate is monomorphic and is made of small to medium-sized round cells with scant cytoplasm, and inconspicuous nucleoli. The tumor infiltrate shows a prominent epitheliotropic pattern but lacks inflammatory background. It is usually CD3+, CD4−/CD8+, CD56+, MATK+, expresses cytotoxic molecules, and often lacks CD5, indicating a possible γδ T-cell derivation. TCRγ is often rearranged, and gains at 9q34.3 as well as STAT5b mutations are frequently observed. The behavior and survival are similar to that of EATL [12,13,15,16,17].

ITCLDGT is a clonal proliferative disorder of small CD8+ intestinal T lymphocytes that lacks destruction of the bowel mucosa, with a typical lymphoid cell infiltration of the lamina propria with displacement of mucosal glands and epithelium sparing, but may be unresponsive to chemotherapy and progress to a frank aggressive ITCL [6,18,19].

ITCLDGT is limited to the gastrointestinal tract. Presenting symptoms include abdominal pain, weight loss, and diarrhea with a chronic and relapsing clinical course. Proliferative index is low and patients experience a prolonged survival [10,18,20].

iNKLPD is a recently described entity, previously considered a reactive process because of its self-regressive lesions. It may also involve gallbladder and vagina and it is characterized by an atypical infiltrate of medium-sized cells with NK/T-cell lymphoma phenotype (cytoplasmic CD3+, CD7+, CD56+, expression of cytotoxic molecules; and CD4−, CD5−, CD20−), but lacking EBV infection [6,21,22,23,24]. From a molecular point of view, iNKLPD seems to have recurrent somatic JAK3 gene mutations [25].

Finally, ITCL-NOS is a basket category used for all the primary ITCL lacking the diagnostic criteria for other ITCL subtypes. It more frequently involves the colon, with a proliferation of cytotoxic TCR-silent lymphocytes, and displays an aggressive clinical behavior [2,4,8].

The literature currently includes a small number of ITCL series from different countries, often focusing on specific biological or molecular features of a single ITCL subtype [11,16,26]. The aim of our observational retrospective study was to depict the complete clinical–pathological profile of a series of 28 patients affected by primary ITCL involving the small bowel diagnosed and treated at the “Istituto di Ricovero e Cura a Carattere Scientifico” (IRCCS) San Matteo Foundation Hospital of Pavia, in Northern Italy, which is a referral center for the diagnosis and treatment of both CD and lymphomas. Thus, we tried to define clinical and histopathological hallmarks of these lymphomas, widening the knowledge on these rare and still partially characterized neoplasms.

## 2. Materials and Methods

### 2.1. Patients

We collected all the GI lymphomas diagnosed at the center between January 2001 and December 2021 and we therefore included in the present study only the cases with a diagnosis of primary ITCL involving the small bowel. Two patients were previously reported by our group [27].

All patients received a histopathological diagnosis of ITCL, with analysis of one or more histological samples and a complete clinical–radiological assessment including computed tomography (CT) and positron electron tomography (PET) at diagnosis. Histopathological diagnosis of ITCL was made according to the 5th edition of WHO classification of hematopoietic tumors [6]. Two experienced hematopathologists (M.L. and M.P.) reviewed all diagnostic slides (i.e., H&E and immunohistochemical stains).

### 2.2. Clinical Data

For each patient we retrieved a pool of clinical data, i.e., sex, age, laboratory tests—blood cell counts, ß2-microglobulin and LDH-, site of involvement, history of CD, symptoms at presentation, gastrointestinal complications—perforation, hemorrhage, fistula, or obstruction, clinical staging, overall survival, treatment, and outcome.

To calculate the overall survival (OS) we considered the time between the initial diagnosis and the death or last follow-up. The median follow-up was 12 moths (IQR 8–17).

We used the Lugano staging system for GI lymphomas [28]. This system distinguishes a stage I lymphoma, with a confined involvement of GI tract—further divided into stage IE1 and IE2 according to the involvement of mucosa and submucosa or muscolaris propria and serosa, respectively; a stage II lymphoma, with extension into abdomen, further classified in II1 with involvement of local lymph nodes, II2 with involvement of distant lymph nodes, and IIE with serosa penetration to involve adjacent organs and tissues; and, finally, a stage IV lymphoma, with disseminated extranodal involvement or supradiaphragmatic nodal involvement.

### 2.3. Histopathological and Molecular Data

Histopathological and molecular data were also collected (i.e., cell features, immunophenotype, level of bowel wall infiltration, presence of necrosis, ulceration, or angiotropism, inflammatory infiltrate, Epstein–Barr virus (EBV) positivity assessed by EBER-ISH, proliferative index, and presence of TCR rearrangement according to BIOMED-2 protocol).

### 2.4. Statistical Analysis

Statistical analysis was performed using R software (version 4.0.3—2020 survival package; R Foundation for Statistical Computing, Wien, Austria). We performed univariate survival analyses for clinical and pathological features through the Kaplan–Meier method, and differences were compared with the log-rank test. For the multivariate survival analysis, a Cox regression model was used. Two-sided *p*-value < 0.05 was considered statistically significant. Given the low sample size, it was not possible to statistically compare clinical, histopathological, and molecular variables among lymphoma subtypes. The study was approved in 2014 by the local ethics committee, and all participants provided written informed consent for the publication of anonymized data. The study is reported in accordance to the STROBE recommendations for quality assurance.

## 3. Results

### 3.1. Clinical Features

Between January 2001 and August 2021, we diagnosed at our center 226 cases of GI lymphoma. B-cell lymphomas accounted for 193 cases, while 33 were T-cell lymphomas, including 28 ITCL, 2 ALCL, 1 ENKTCL, and 2 cases of nodal T-cell lymphomas with secondary intestinal involvement.

We collected 28 cases of primary ITCL, including 17 EATL, 5 MEITL, 3 ITCLDGT, and 3 ITCL-NOS. No cases of iNKLPD were found in our lymphoma series. ITCL represented 12% of all GI tract lymphomas followed in the last 20 years at our institution. Clinical features are summarized in Table 1. Our cohort included 16 males and 12 females, with a median age at diagnosis of 59 years. Most patients were referred for GI and systemic symptoms such as weight loss, diarrhea, fever, or GI complications. Overall, more than 80% (23/28) of patients presented with B-symptoms. At endoscopic, surgical, and pathologic gross examination, ITCL involved a single site in 64.3% of patients, mostly the ileum.

Lymph nodes were involved in 20/28 cases (71%), with localization to distant stations in 3 cases only. Spleen and/or liver were involved in up to 18% of patients (5/28, 3/17 EATL 1/5 MEITL and 1/3 ITCLDGT) while bone marrow was involved in 3/28 cases (10%). Other extraintestinal sites involvement, i.e., lung, pancreas, or bladder, was observed in only three cases. The ITCL was associated with a monoclonal gammopathy of undetermined significance (MGUS) in two cases, and with hepatocellular carcinoma or G1 chondrosarcoma in one patient each. Interestingly, the last patient (diagnosed as ITCLDGT) was also affected by Noonan syndrome. CD was diagnosed in around 70% of our patients (19/28, 15/17 EATL 3/5 MEITL and 1/3 ITCLDGT) 

GI complications (i.e., perforation, hemorrhage, fistula, or obstruction) were observed in more than one-third of cases (10/28). The clinical stage, according to Lugano clinical staging system, was IV (disseminated) in 40% of cases.

LDH was elevated in 25/28 (89%) patients. Nevertheless, no patient showed a marked elevation (i.e., over fourfold increase from the upper limit of 220 U/L) of the enzyme. Similarly, β2-microglobulin was elevated in all patients (more than 2500 mcg/L), but above 5500 mcg/L in 11/28 patients only. The blood cell counts are reported in Table 1.

The median overall survival (OS) was 12 months. Only 3/28 patients (10.7%) were still alive at the last follow-up and with partial response (>50% of lesion decrease) to chemotherapy, while the remaining died as a consequence of the lymphoma.

We could retrieve information regarding treatment schedules in 19/28 cases. These patients were mainly treated according either to CHOP (cyclophosphamide, doxorubicin, vincristine, prednisone) for the 68% or CHOEP (CHOP plus etoposide) for the 21% schemes. Only two EATL patients were treated with stem cell transplantation: one of them died after 12 months while the other was alive at the last follow-up. Two patients died before receiving any treatment.

### 3.2. Univariate and Multivariate Survival Analysis

We performed univariate survival analysis for clinical and histopathological variables.

**Figure 1:**
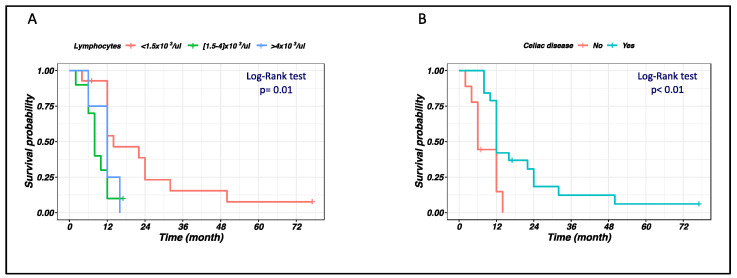
Kaplan–Meier curves showing difference in survival in patients with intestinal T-cell lymphomas (ITCL) involving the small bowel, according to clinical variables. Differences were compared with the log-rank test, and a two-sided *p*-value < 0.05 was considered statistically significant. (**A**) Patients with low lymphocyte counts show better survival than those with marked lymphocytosis (lymphocytes > 4 × 10^3^/μL); (**B**) Patients with no history of celiac disease (CD) show worse survival than those with previous CD diagnosis.

As shown in the Kaplan–Meier plots in Figure 1, lymphocyte count and history of CD are the clinical variables that showed a significant impact on survival. In detail, a lymphocytosis (namely, a lymphocyte count > 4 × 10^3^/μL) and the absence of CD history were associated with a worse outcome for patients with ITCL. All the other clinical variables taken into account and reported in Table 1 were not found to impact on survival at log-rank test.

When evaluating the effect of therapy (CHOP vs. CHOEP) on survival, no statistical difference was found with the log-rank rest (*p* = 0.34).

**Figure 2:**
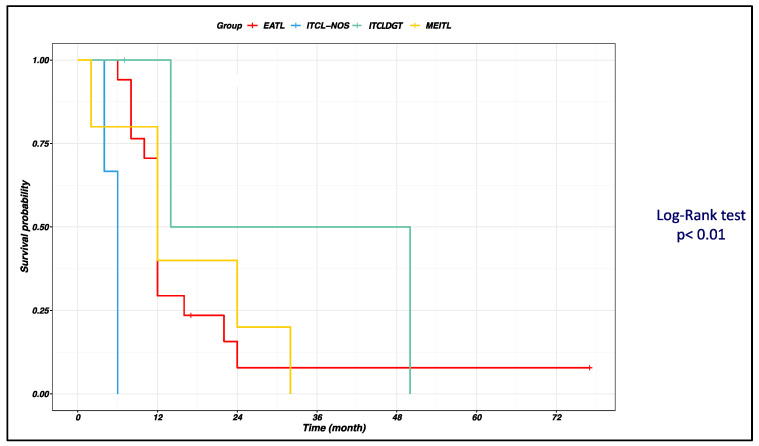
Kaplan–Meier curves showing difference in survival in patients with intestinal T-cell lymphomas (ITCL) involving the small bowel, according to histopathological subtypes. Differences between the four curves were evaluated with the log-rank test and a two-sided *p*-value < 0.01 was found, showing that the variant with the worst prognosis was intestinal T-cell lymphoma not otherwise specified (ITCL-NOS), while the one with the best prognosis was the indolent T-cell lymphoproliferative disorder of the gastrointestinal tract (ITCLDGT).

Taking into account the impact on survival of different histologic subtypes, univariate analysis comparing the four survival curves (EATL vs. METL vs. ITCLDGT vs. ITCL-NOS) showed that ITCL-NOS had the worst survival probability while ITCLDGT had the best (Figure 2).

Finally, multivariate analysis confirmed the role of significant variables at univariate, with the addition of Lugano clinical stage. In detail, a Lugano stage of II or greater was correlated with a worse prognosis for patients with ITCL.

### 3.3. Specific Clinical, Histopathological, and Molecular Features of ITCL Subtypes

#### 3.3.1. EATL

EATL was the most frequent ITCL observed in our center (64% of cases) and showed the lower M:F ratio. EATL more frequently displayed a disseminated disease and some clinical peculiarities compared to other ITCL. The most frequently involved site was the distal small intestine, with ileum or jejunum being involved in over half of patients. Most EATL cases (15/17, 88%) had an associated CD, diagnosed before the occurrence of lymphoma (6/17 cases) or at the same time of the tumor diagnosis (7/17 cases). More in detail, a diagnosis of refractory celiac disease (RCD) was made in 6 out of 17 patients. In the two remaining cases, a clear diagnosis of CD could not be established due to EATL severity leading to death and early work-up termination.

Clinically, EATL displayed the highest frequency of B-symptoms and organomegaly. Furthermore, it was the only ITCL type to involve the bone marrow in our series. Consistently, 59% of cases were classified as clinical stage III or IV. All but one patient who displayed GI complications in our ITCL cohort were diagnosed as EATL.

The median OS of EATL was 12 months.

**Figure 3:**
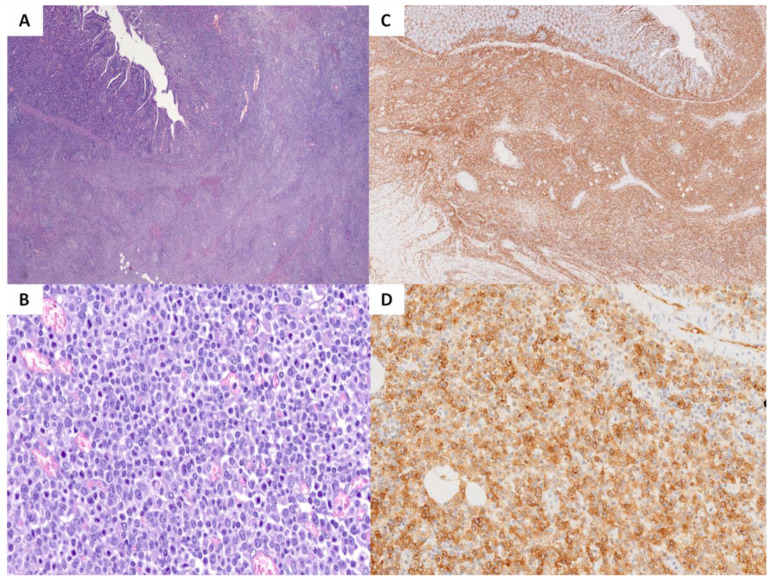
Enteropathy-associated T-cell lymphoma (EATL). Hematoxylin–eosin staining ((**A**), 20×) shows a diffuse polymorphic ((**B**), 400×) proliferation of T-cells frequently expressing CD3 ((**C**), 20×) and, in most of our cases, CD30 ((**D**), 200×).

From a histopathological point of view, diagnosis of EATL was not significantly associated with a specific immunophenotype, cell size, or neoplastic infiltrate’s features such as necrosis. Nevertheless, EATL was generally characterized by a polymorphic proliferation of medium to large-sized cytotoxic T-cells frequently expressing CD3 and CD7, double-negative for CD4 and CD8, and staining for CD30 more in than half of cases (11/17) (Figure 3). Villous atrophy was present in the uninvolved mucosa beside the tumor in seven cases. The presence of eosinophils in the neoplastic infiltrate was observed in 61% of patients. The median proliferation index was 60%, and TCRγ rearrangement was detected in all the analyzed cases (7/7) (Table 2).

#### 3.3.2. MEITL

Compared to other ITCL, in our cohort, MEITL was slightly more frequent in males and had a median age at diagnosis of 60 years. The lymphoma involved only the small intestine, with multiple localizations in a single case. B-symptoms were observed in 40% of cases, while organomegaly was present in one single patient. Regional lymph node involvement was present in 80% of cases. Interestingly, three of our MEITL patients received a diagnosis of CD. A total of 60% of patients displayed a clinical stage I or II.

All patients died within a median of 12 months.

From a histopathological perspective, the tumor infiltrate consisted either of small to medium or large monomorphic cells. The presence of angiotropism was observed in 80% of cases. The neoplastic cells were more often CD3^+^, CD2^+^, CD7^+^, and TIA-1^+^. All patients were positive for CD56 and 80% of them were CD8+. The CD8 seemed to be more typical of this subtype compared to other subtypes of ITCL, in which it was expressed in only one-third of the cases. A median of 55% of cells were positive for Ki67. A single patient was analyzed and tested positive for TCRγ rearrangements as previously described (Table 2) [27].

#### 3.3.3. ITCLDGT

We observed three cases of ITCLDGT. They were two males and one female, with a median age at presentation of 68 years. All cases involved the stomach and duodenum. Serum β2-microglobulin was increased (median value 5860 mcg/L) and B-symptoms were present in all cases. Moreover, all cases showed involvement of locoregional lymph nodes. Nevertheless, the lymphoma showed no organomegaly, involvement of bone marrow, or distant lymph nodes. Two patients received a first diagnosis of EATL and were treated with CHOP without success. The diagnosis of ITCLDGT was made after a review of the biopsies performed at initial presentation, with a diagnostic delay of 2 months for one case and 4 years for the other. All the ITCLDGT patients of our cohort were at stage IV of Lugano and the OS was of 14 months in median. Only one patient was alive at the last follow-up. This subtype showed the best survival probability, when compared to other subtypes, at univariate and multivariate analysis.

**Figure 4:**
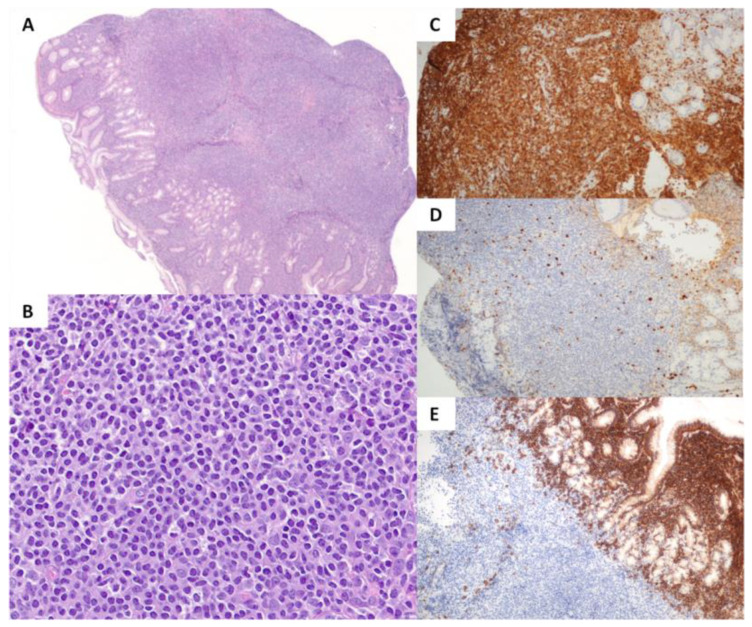
Indolent T-cell lymphoproliferative disorder of the gastrointestinal tract (ITCLDGT). Hematoxylin–eosin staining ((**A**), 20×) shows a proliferation of small to medium-sized ((**B**), 400×) T-cells CD3+ ((**C**), 100×) and with a low proliferative index ((**D**), 100×). In 2/3 cases, the background infiltrate was rich in CD138+ plasma cells ((**E**), 100×).

The lymphoma cells were small to medium-sized and resulted as positive, in all patients, for CD3, CD2, CD7, and TIA1 and negative for ALK, CD56, CD57, granzyme-B, and Perforin. Two cases were CD4−/CD8+ and one CD4+/CD8−. CD5 and βF1 were positive in one case each. Ki67 proliferative index was low (5–15%). No bystander eosinophils, angiotropism, or necrosis was observed. Interestingly, in two out of three ITCLDGT cases, the infiltrate was rich in plasma cells (Figure 4 and Table 2). One of our cases was also diagnosed with Noonan syndrome and had previously developed a G1 chondrosarcoma treated with surgery only.

#### 3.3.4. ITCL-NOS

Three patients received a diagnosis of ITCL-NOS, as it was not possible to make an alternative diagnosis according to the 2022 WHO classification criteria. These were two males and one female with a median of 65 years at diagnosis. They had a more distally localized lymphoma, comparing to other categories, and 2/3 patients displayed multiple sites of involvement over the GI tract. They all presented with B-symptoms and with a high serum concentration of β2-microglobulin (median value 6700 mcg/L). They all had lymph node involvement, including distant stations. Two out of three patients presented with a clinical stage IV. All patients died within a median of 5 months, and at univariate and multivariate analysis, the ITCL-NOS showed the worst survival probability.

At histopathological examination, two cases displayed infiltrates made by small to medium-sized cells, and just one by large cells. In two cases, the lymphoma cells expressed CD3, CD7, and Granzyme-B. No cases tested positive for CD4, CD20, CD56, or CD57. Interestingly, two patients displayed necrosis and bystander eosinophils, similarly to our EATL cases. ITCL-NOS showed the higher median proliferative index (Ki67), equal to 60% (Table 2).

## 4. Discussion

ITCL with a primary involvement of the small bowel are a group of rare primary extranodal lymphomas possibly arising from innate intraepithelial intestinal resident T-cells. Even though in most cases ITCL behaves as very aggressive neoplasm, the clinical, histopathological, and molecular features as well as the postulated cell-of-origin differ among the various entities. Moreover, the incidence of the different ITCLs highly depends on the geographic area, as a consequence of the higher incidence of CD in Western countries, and the higher incidence of lymphoma of NK (EBV-driven) or γδ T-cell in Asia and Central and South America [26].

The proportions of different ITCL subtypes in our article reflect the Caucasian origin of all our patients. Indeed, our most frequent diagnosis was of EATL, in most cases being associated with CD. The epidemiological, clinical, and histopathological data of all our patients were in line with the current knowledge. EATL develops in the late adulthood with a slight male predominance and presents with persistent GI and B-symptoms. EATL generally involves the distal small intestine (jejunum and ileum) and may be preceded by clinical and histological evidence of RCD. EATL seems to derive from so-called “type A” innate intraepithelial T-cells [29] carrying an alpha/beta TCR, whereas MEITL may derive from either alpha–beta or gamma–delta intraepithelial T-lymphocytes. In our series, we could detect beta-F1 expression only in a minority of cases (21% of the cases, irrespective of the histologic subtype). Although the sensitivity of beta-F1 immunohistochemical analysis alone may not be sufficient to establish a gamma–delta histogenesis, our data suggest the opportunity to explore more deeply the possibility that even a significant fraction of ITCL may derive from gamma–delta T-cells and their possible prognostic significance.

EATLs frequently harbor cytogenetic alterations with loss of function of *CDKN2A* (9p loss/9p21 LOH) and mutation in *SETD2*, *STAT5b*, or *JAK1*/*JAK3* genes [30]. They generally show a CD3+, CD103+, CD30+/−, double CD4/CD8 negative, or CD8+ cytotoxic phenotype. In our study, CD30 expression was found almost exclusively in EATL, suggesting a possible diagnostic utility of this marker.

Extraintestinal localizations are more common compared to other ITCL and involve both lymphatic and non-lymphatic organs. Interestingly, our data show a high incidence of splenic, and especially liver, involvement.

Most patients with EATL had a concurrent or previous diagnosis of CD or RCD. This finding is in keeping with other studies, in which a variable association between EATL and CD (between 38 and 100%) was described [31,32]. We cannot exclude that the rare cases (2/17) without a definite diagnosis of CD may, in fact, be celiac patients where the aggressive presentation and course of EATL prevented a complete diagnostic work-up to confirm CD diagnosis.

The majority of EATL patients received chemotherapy, respectively, with CHOP or CHOEP schemes. Only two EATL patients were treated with stem cell transplant, and one of them was still alive at the last follow-up. Although there are no standardized treatment protocols and the optimal therapy remains unclear, CHOP and CHOEP protocols represent the best choice according to experts [33,34]. However, the results of such therapy on survival of EATL patients are unsatisfying and few alternative strategies are currently available [35]. Brentuximab, an anti-CD30 antibody, appears to be a promising therapy in cases with CD30 expression [36]. Therefore, assessment of CD30 expression seems to be relevant not only to characterize the disease but also for therapeutic purposes. In our study, CD30 appears to be closely associated with EATL.

We do not have reliable prognostic tools for this type of lymphoma, and the disease stage and International Prognostic Index (IPI) are not useful in predicting survival. Recently a new validated prognostic model, EATL prognostic index (EPI), was proposed to predict survival outcome in EATL and to select patients for new therapeutic strategies [37]. This new model distinguishes three risk groups (high risk, intermediate risk, and low risk) combining IPI score with the presence of B-symptoms. Our study showed how other variables, such as peripheral lymphocytosis, diagnosis of CD, Lugano clinical stage, and histological subtype, may be considered for a more accurate stratification of ITCL patients. In particular, peripheral lymphocytosis seems to be associated with a worse prognosis; however, the lymphocyte phenotype characterization would be necessary to draw firm conclusions and define its biological significance.

It seems reasonable that only the combination of clinicopathologic variables with molecular and genetic parameters can permit proper stratification of ITCL patients, favoring a more personalized therapeutic approach, keeping in mind that these conditions remain very aggressive with poor median OS (12 months).

MEITL was the second-most common diagnosis in our cohort. Despite the clinical presentation overlap with EATL, histopathological analysis revealed some peculiarities. In fact, we found a positive correlation between the diagnosis of MEITL, the presence of angiotropism, and the expression of CD56. Angiotropism was previously described as a frequent feature in EATL, while we observed it in only 20% of our EATL cases compared to 80% of MEITL. On the contrary, CD56 expression in MEITL has been widely described, but it was never proved to be a specific marker of MEITL diagnosis.

MEITL seems to derive from “type B” intraepithelial effector memory T-cells, frequently expressing CD8α, together with CD3, CD7, CD56, and cytotoxicity markers. Moreover, MATK has been recently described as novel specific markers [16,38]. On a genetic level, these lymphomas show frequent gains of 8q24 (including *MYC* gene) and recurrent mutations of *STAT5b*, *JAK3*, *GNAI2*, and *SETD2*. Intriguingly, the presence of (1) epitheliotropic pattern and angiotropism, (2) the frequent expression of CD8 and cytotoxic markers, (3) the evidence of recurrent mutation in *JAK2* and *STAT5b* genes, (4) the involvement of epithelial interfaces, and (5) the very aggressive clinical behavior are common denominators between MEITL and aggressive epidermotropic cytotoxic T-cell lymphoma, a rare lymphoma involving the skin. Thus, it should be of interest to better understand the connections between these two rare lymphomas.

We report how, in our case series, three out of five patients with MEITL had a diagnosis of CD. Even though both EATL and MEITL have a male predominance, two out of three of our MEITL patients with CD diagnosis were females. Clinically, they showed a clinical stage III in two cases and a clinical stage II in one, and they all died of disease. From a pathological point of view, all three of them showed a typical MEITL histology with the presence of a monomorphic infiltrate of CD3+, CD7+, CD56+, and CD5− cells with expression of cytotoxic molecules. Thus, despite the small number of cases, this finding seems to confirm our previous work showing that EATL and MEITL share some clinical and pathologic features and some chromosomal abnormalities that could justify, in the Caucasian population, the association of CD with both lymphoma subtypes [27]. However, further studies are required to better understand this possible association. Despite the little knowledge on this lymphoma and the recent evidence of their molecular heterogeneity, their phenotype is relatively helpful to address diagnosis given their almost uniform expression of CD56, as we showed in our results [39].

Our cases of ITCLDGT displayed many interesting features. Firstly, they all involved the stomach. Secondly, they also showed an association with some histopathological features, some of which have never previously been reported (the presence of many plasma cells within neoplastic infiltrate). It was also of great importance to notice that two out of three patients were initially diagnosed as EATL and then treated accordingly. This highlights that ITCLDGT diagnosis may be challenging, giving the higher frequency of aggressive ITCL and the absence of useful diagnostic markers. For this reason, considering the low number of our ITCLDGT patients, the significant features associated with this diagnosis in our study needs to be considered and validated in larger series. Recently, a specific *STAT3-JAK2* gene fusion was described in CD4+ ITCLDGT [40,41]. We had a single CD4+ case; still, we could not perform molecular analysis due to scarce biological material.

Despite the longer survival described for this subtype of lymphoma, our three patients showed a median survival of 14 months, only slightly better than EATL. All three patients were diagnosed at an advanced stage and their death was associated with the course of the disease (not with complications or other causes).

One patient with ITCLDGT had a diagnosis of CD, representing, to the best of our knowledge, the first described case of association. The heterogeneity of this entity could explain a possible association with CD, but further studies are needed to confirm this possibility.

Similarly to ITCLDGT, ITCL-NOS is an exceedingly rare lymphoma of postulated peripheral T-cell origin (but possibly arising from innate T-resident lymphocytes). In our study, these lymphomas localized in the distal small bowel as well as in the large bowel, and behaved very aggressively, similarly to EATL or MEITL. Our ITCL-NOS cases did not display any specific clinical or histopathological features. This may be due to the fact that this is a basket category reflecting a still-unraveled biological heterogeneity.

All in all, our study showed some limitations. First, the small number of cases, due to the rarity of the diseases, might have impaired the statistical analysis results. In particular, the aggressiveness of these lymphomas, resulting in high mortality, may have affected the survival analysis. Secondly, this retrospective study analyzed cases collected over a long time span, therefore we could not obtain complete data for all of our patients. Particularly, we could not biologically characterize peripheral lymphocytosis. Lastly, as previously reported, the association between CD and ITCL could have been underestimated, because the rapidly fatal course of these lymphomas may prevent, in some cases, achieving a complete work-up for CD diagnosis.

Despite these limitations, our study is one of the largest ITCL case series reported in the current literature; thus, it may help to deepen our knowledge and to improve the management of these rare and only partially characterized lymphomas.

## 5. Conclusions

In conclusion, primary ITCLs involving the small bowel are rare and frequently aggressive conditions, with many common clinical features but some different histopathological and molecular findings. Further studies on a larger number of patients are needed to better characterize these rare conditions, achieve better prognostic stratification, and improve the therapeutic approach.

## Figures and Tables

**Table 1:** Clinical presentation features of patients included in this study with primary ITCL involving the small bowel.

	All ITCL	EATL	MEITL	ITCL-NOS	ITCLDGT
**Patients n. (%)**	28/28 (100)	17/28 (60.7)	5/28 (17.9)	3/28 (10.7)	3/28 (10.7)
**Age median [range]**	59 [38–74]	55 [44–74]	60 [57–71]	65 [38–71]	68 [45–71]
**M/F (ratio)**	16/12 (1.3:1)	9/8 (1.1:1)	3/2 (1.5:1)	2/1 (2:1)	2/1 (2:1)
**Blood cell counts median [range]**					
**Hb g/dL**	10.4 [6.4–14.6]	10.2 [7.4–13.5]	10.6 [6.4–14.6]	9.8 [8.8–12.2]	11.6 [11.3–13.1]
**Neutrophils × 10^3^/mmc**	5.3 [1–19.5]	6.1 [1–19.5]	3.4 [1.1–8.9]	5.2 [4.6–10.2]	3.7 [3.6–6.4]
**Lymphocytes × 10^3^/mmc**	1.5 [0.1–4.8]	1.8 [0.1–4.8]	1.4 [1.1–3.4]	1.5 [0.2–4.2]	0.8 [0.6–1.3]
**Lymphocytes < 1.5 × 10^3^/mmc** **n. (%)**	14 (50)	7 (41.1)	3 (60)	1 (33.3)	3 (100)
**Lymphocytes 1.5–4 × 10^3^/mmc** **n. (%)**	10 (35.7)	7 (41.1)	2 (40)	1 (33.3)	0
**Lymphocytes > 4 × 10^3^/mmc** **n. (%)**	4 (14.3)	3 (17.6)	0	1 (33.3)	0
**LDH median [range] U/L**	295 [210–540]	300 [220–440]	310 [260–410]	270 [210–360]	270 [141–540]
**β2MG median [range] mcg/L**	4505 [2780–7800]	4120 [2780–7800]	4005 [2955–6700]	6700 [2900–6700]	5860 [2765–6450]
**Multiple sites n. (%)**	10 (35.7)	4 (23.5)	1 (20)	2 (66.7)	3 (100)
**Celiac disease n. (%)**	19 (67.9)	15 (88.2)	3 (60)	0	1 (33.3)
**GI complications n. (%)**	10 (35.7)	9 (52.9)	1 (20)	0	0
**Lugano stage I n. (%)**	3 (10.7)	2 (11.8)	0	1 (33.3)	0
**Lugano stage II n. (%)**	8 (28.6)	5 (29.4)	3 (60)	0	0
**Lugano stage III n. (%)**	6 (21.4)	3 (17.6)	2 (40)	1 (33.3)	0
**Lugano stage IV n. (%)**	11 (39.2)	7 (41.2)	0	1 (33.3)	3 (100)
**Median overall survival (months) [range]**	12 [2–77]	12 [6–77]	12 [2–32]	5 [4–6]	14 [7–50]
**Death n. at follow up (%)**	25 (89.3)	15 (88.2)	5 (100)	3 (100)	2 (66.7)

Abbreviations: β2MG: beta2-microglobulin; EATL, enteropathy-associated T-cell lymphoma; F, female; GI, gastrointestinal; Hb, hemoglobin; ITCL, intestinal T-cell lymphomas; NOS, not otherwise specified; ITCLDGT, indolent T-cell lymphoproliferative disorder of the gastrointestinal tract; LDH, lactate dehydrogenase; M, male; MEITL, monomorphic epitheliotropic intestinal T-cell lymphoma; PLT, platelet; WBC, white blood cell.

**Table 2:** Histological features of patients included in this study with primary ITCL involving the small bowel.

	All ITCL	EATL	MEITL	ITCL-NOS	ITCLDGT
**Patients n. (%)**	28/28 (100)	17/28 (66.7)	5/28 (17.9)	3/27 (10.7)	3/28 (10.7)
**Ulceration n. (%)**	19 (67.9)	12 (70.6)	5 (100)	0 (0)	1 (33.3)
**Angiotropism n. (%)**	8 (28.6)	4 (23.5)	4 (80)	0 (0)	0 (0)
**Necrosis n. (%)**	12 (42.9)	10 (58.8)	0 (0)	2 (66.7)	0 (0)
**Infiltration n. (%)**					
**Submucosa**	1 (3.6)	0 (0)	0 (0)	1 (33.3)	0 (0)
**Muscularis**	1 (3.6)	1 (5.9)	0 (0)	0 (0)	0 (0)
**Perivisceral fat**	14 (50)	7 (41.2)	5 (100)	2 (66.7)	0 (0)
**Serosa**	2 (7.1)	3 (17.6)	0 (0)	0 (0)	0 (0)
**Cell size n. (%)**					
**Small to medium size**	11 (39.3)	3 (17.6)	2 (40)	2 (66.7)	3 (100)
**Medium to large size**	17 (60.7)	14 (82.4)	3 (60)	1 (33.3)	0 (0)
**Immunophenotype** **n. positive cases (%)**					
**ALK**	0 (0)	0 (0)	0 (0)	n.d.	0 (0)
**CD2**	15 (53.6)	6 (35.3)	3 (60)	1 (33.3)	3 (100)
**CD3**	27 (96.4)	16 (94.1)	5 (100)	3 (100)	3 (100)
**CD4**	2 (7.1)	1 (5.9)	0 (0)	0 (0)	1 (33.3)
**CD5**	6 (21.4)	4 (23.5)	0 (0)	1 (33.3)	1 (33.3)
**CD7**	8 (28.6)	13 (76.5)	5 (100)	2 (66.7)	3 (100)
**CD8**	12 (42.9)	5 (29.4)	4 (80)	1 (33.3)	2 (66.7)
**CD20**	0 (0)	0 (0)	0 (0)	0 (0)	0 (0)
**CD30**	12 (42.9)	11 (64.7)	0 (0)	1 (33.3)	0 (0)
**CD56**	7 (25)	2 (11.8)	5 (100)	0 (0)	0 (0)
**CD57**	1 (3.6)	0 (0)	1 (20)	0 (0)	0 (0)
**Granzyme-B**	11 (39.3)	7 (41.2)	2 (40)	2 (66.7)	0 (0)
**Perforin**	14 (50)	12 (70.6)	1 (20)	1 (33.3)	0 (0)
**TIA-1**	18 (64.3)	11 (64.7)	5 (100)	1 (33.3)	3 (100)
**β-F1**	6 (21.4)	3 (17.6)	1 (20)	1 (33.3)	1 (33.3)
**EBV-ISH n. positive cases (%)**	1 (3.6)	0 (0)	0 (0)	0 (0)	1 (33.3)
**Ki67 median [range]**	50 [5–80]	60 [25–80]	55 [40–75]	60 [40–80]	10 [5–15]
**TCRγ rearrangement n. (%)**	9/9 (100)	7/7 (100)	1/1 (100)	n.d.	2 (66.7)
**Eosinophils in ITCL infiltrate n. (%)**	13 (46.4)	10 (58.8)	0 (0)	2 (66.7)	1 (33.3)
IELS > 40/100 enterocytes n. (%) *	10 (35.7)	6 (35.3)	3 (60)	n.d.	1 (33.3)
Villous atrophy n. (%) *	8 (28.6)	7 (41.2)	1 (20)	0 (0)	0 (0)

* In non-neoplastic mucosa. Abbreviations: EATL, enteropathy-associated T-cell lymphoma; EBV, Epstein–Barr virus; IELS, intraepithelial lymphocytes; ISH, in situ hybridization; ITCL, intestinal T-cell lymphomas; ITCLDGT, indolent T-cell lymphoproliferative disorder of the gastrointestinal tract; MEITL, monomorphic epitheliotropic intestinal T-cell lymphoma; n.d., not determined; NOS, not otherwise specified; TCR, T-cell receptor.

## Data Availability

The data presented in this study are available on request from the corresponding author.
